# From Samples to Germline and Somatic Sequence Variation: A Focus on Next-Generation Sequencing in Melanoma Research

**DOI:** 10.3390/life12111939

**Published:** 2022-11-21

**Authors:** Adrián Muñoz-Barrera, Luis A. Rubio-Rodríguez, Ana Díaz-de Usera, David Jáspez, José M. Lorenzo-Salazar, Rafaela González-Montelongo, Víctor García-Olivares, Carlos Flores

**Affiliations:** 1 Genomics Division, Instituto Tecnológico y de Energías Renovables (ITER), 38600 Santa Cruz de Tenerife, Spain; 2 Research Unit, Hospital Universitario Nuestra Señora de Candelaria, 38010 Santa Cruz de Tenerife, Spain; 3 CIBER de Enfermedades Respiratorias, Instituto de Salud Carlos III, 28029 Madrid, Spain; 4 Facultad de Ciencias de la Salud, Universidad Fernando de Pessoa Canarias, 35450 Las Palmas de Gran Canaria, Spain

**Keywords:** cancer genomics, melanoma, next-generation sequencing, third-generation sequencing, nanopore, bioinformatic workflows, pipeline, clinical genomics, personalized medicine

## Abstract

Next-generation sequencing (NGS) applications have flourished in the last decade, permitting the identification of cancer driver genes and profoundly expanding the possibilities of genomic studies of cancer, including melanoma. Here we aimed to present a technical review across many of the methodological approaches brought by the use of NGS applications with a focus on assessing germline and somatic sequence variation. We provide cautionary notes and discuss key technical details involved in library preparation, the most common problems with the samples, and guidance to circumvent them. We also provide an overview of the sequence-based methods for cancer genomics, exposing the pros and cons of targeted sequencing vs. exome or whole-genome sequencing (WGS), the fundamentals of the most common commercial platforms, and a comparison of throughputs and key applications. Details of the steps and the main software involved in the bioinformatics processing of the sequencing results, from preprocessing to variant prioritization and filtering, are also provided in the context of the full spectrum of genetic variation (SNVs, indels, CNVs, structural variation, and gene fusions). Finally, we put the emphasis on selected bioinformatic pipelines behind (a) short-read WGS identification of small germline and somatic variants, (b) detection of gene fusions from transcriptomes, and (c) de novo assembly of genomes from long-read WGS data. Overall, we provide comprehensive guidance across the main methodological procedures involved in obtaining sequencing results for the most common short- and long-read NGS platforms, highlighting key applications in melanoma research.

## 1. Introduction

Cutaneous melanoma is the major culprit in skin cancer-related mortality, as it is a highly aggressive skin tumor with the highest mutation load among tumors [1]. As with any other type of cancer, cutaneous melanoma could have a somatic (i.e., sporadic) or a germinal (i.e., familial) origin. The first one is the most common form, explaining ~90% of all melanoma cases, and it is caused by weak- or moderate-risk somatic mutations [2,3]. These could explain why sporadic cutaneous melanoma shows a clear relationship with risk factors such as the presence of naevi [4,5,6], exposure to UV irradiation [7,8,9,10], and with polygenic factors such as fair skin [11,12], among others. In addition, the most frequent and well-known genetic alterations occurring in melanoma are linked to the *BRAF*, *NRAS*, *KIT*, and *NF1* genes [13,14,15,16]. The familial form of cutaneous melanoma (i.e., in families with at least another relative affected) has an incidence of ~8% of the cases. In this respect, the vast majority of the highly penetrant germline mutations mainly affect the *CDKN2A* and *CDK4* genes [17].

The advent and adoption of next-generation sequencing (NGS) technologies have accelerated the development of human genomics and personalized medicine, allowing us to study the role of both germline and somatic mutations more precisely in disease. This has facilitated the increase in knowledge of most cancer types, including melanoma, through genomic, transcriptomic, and epigenomic approaches. The decrease in costs and the increase in coverage of targeted gene panels, whole-exome (WES), whole-genome (WGS), and transcriptome (RNA-Seq) sequencing applications offer the possibility of rapidly improving clinical studies, triggering novel and more comprehensive analyses in cancer research [18]. Recent advances in long-read sequencing or third-generation sequencing (TGS), such as those provided by Pacific Biosciences (PacBio) and Oxford Nanopore Technologies (ONT), are nowadays booming because of the facilitation of studies of somatic mutations that affect large and complex regions of the genome that would be difficult to analyze otherwise with the more standard short-read sequencing approaches [19,20].

This review provides a summary of the technical details involved in library preparation for the sequencing process, describing different alternatives for assessing tumor tissues, as well as outlining the NGS and TGS technologies and their use in the study of germline and somatic variation in cancer, with a focus on melanoma. For that, we have performed a systematic review of the most recent literature in search of studies that have applied high-throughput sequencing methods in cancer studies, especially those focused on cutaneous melanoma. Only studies in humans and written in English were included in this assessment. The search was performed in the NCBI PubMed from May to October 2022, using the following terms: “melanoma”, “cutaneous melanoma”, “cancer genomics”, “DNA library preparation”, “RNA library preparation”, “next-generation sequencing melanoma”, “somatic bioinformatics”, “somatic pipelines”, “somatic structural variation”, “structural variation in cancer”, “NGS quality control”, “kinship estimation”, “transcriptomics gene-fusion”, “long reads cancer genomics”. Besides this, and given that we aimed to review many steps of the methodology, we also revised articles that were published after 2010 until October 2022. For obvious reasons, this excludes the specific literature reviewed that focuses on the classical sequencing approaches or previous technical methods. A detailed description of the steps involved in selected bioinformatic workflows for short and structural variant discovery is also provided, both for short and long-read technologies, highlighting scripting languages and pipeline editors considered standards in the field and detailing the most commonly used tools and databases that are needed to functionally annotate and classify the discovered variants.

## 2. DNA Libraries

In the context of cutaneous melanoma, sequencing techniques used have included, among others, targeted sequencing (focusing on specific regions of the genome when prior information is available), WES (limited to the gene-coding regions and alike), and WGS (to detect alterations in the coding and non-coding regions of the genome). Studies leveraging WES and WGS have helped to identify genes that are important for melanoma pathogenesis and, for example, for improving the classification of the different molecular subtypes of melanoma [21].

The typical NGS workflow comprises different steps, from nucleic acids extraction to variant annotation. The process generally begins with converting nucleic acids (RNA or DNA) from biological samples to a biomaterial compatible with the sequencing system intended for the study. This first step is referred to as library preparation—a library is a set of DNA fragments with attached adapters—and it is one of the most important steps, having key biological and bioinformatics implications [22]. Some of the main factors to consider in obtaining high-quality sequencing libraries are the quantity and integrity of the starting material, and the application to be performed. One of the difficulties when working with melanoma-affected tissues is that the starting material is degraded or of a limited amount. Most excised melanoma lesions are small, from 1 to 2 mm in thickness, and the entire tumor requires formalin-fixed and paraffin wax embedded (FFPE) for diagnosis by histopathologic examination, typically precluding the availability of the frozen tissue, which is more optimal for research. DNA and RNA extraction methods for FFPE tissues vary in the quality and quantity of the resulting material, which may impact the performance of downstream assays. Fixation protocols vary between laboratories. The type of fixative, temperature, pH, chemical crosslinking, or exposure time to formalin and how they are handled contribute to potential nucleic acid damage [23,24].

DNA from FFPE tumor tissues is fragmented, often in low concentration. In melanoma research, the purified DNA can also be contaminated with the pigment melanin, which inhibits polymerase activity [25]. After fixation, DNA fragmentation also changes and increases over time [26], even under certain storage conditions [27]. Usually, the amount of damage in FFPE tissue correlates with the age of the sample. The use of melanoma FFPE samples in amplicon-based NGS panels has shown that storage time was the most critical variable that influenced sample viability for library construction. In this case, the incorporation of quality control (QC) steps and a measure of the DNA integrity (DIN, DNA Integrity Number) helped refine the rate of conversion from samples to NGS results, and particularly to identify which of the oldest samples could be used in the study [28]. Moreover, formalin-induced deamination can lead to artifactual cytosine (C) to thymine (T) and guanine (G) to adenine (A) (C:G > T:A) mutation calls. The proportion of deaminated C bases by formalin fixation is low, generating false low-frequency single nucleotide variants (SNVs). These low-frequency mutations also occur naturally in the tumor process and may be of clinical importance. Therefore, it is essential to repair the deamination in FFPE DNA samples before continuing the rest of the process [29].

Library preparation methods are of the utmost importance when only a small amount of starting material is available and clinical samples are precious. The starting material is generally isolated double-stranded genomic DNA, and the DNA is enzymatically or physically fragmented, followed by end-repair and adapter ligation. Adaptor ligation is followed by size selection to remove free adapters and select the libraries in the desired size range. PCR amplification could also be performed in the resulting selection to obtain enough template DNA for accurate quantification and to further enrich the libraries. However, the amplification step is known to introduce some bias together with fragmentation and size selection [30]. Alternatively, PCR can also be used to add the adapter sequence using tailed primers, which generate molecules with all the elements necessary for sequencing.

Moreover, obtaining the highest possible level of sequence complexity in an NGS library is crucial, as this will reduce the amount of bias. Library complexity refers to the number of unique DNA fragments that are present, i.e., the library should reflect the starting material as closely as possible. The loss of complexity, derived from using PCR, increases the number of duplicate reads. Moreover, shorter fragments are less specific in the bioinformatic alignment against the genome reference step and, thus, decrease the complexity of a sample. In addition to the above PCR considerations, the presence of melanin could inhibit the reaction [31] by forming reversible complexes with DNA polymerase [25]. Additional treatments that allow the proper use of PCR have been described, such as the addition of bovine serum albumin (BSA), DNA dilutions, and DNA purifications with the NucleoSpin^®^ gDNA Clean-up XS kit [32]. A study supported that centrifugation combined with the OneStep™ PCR Inhibitor Removal Kit (Zymo Research Corp, Irvine, CA, USA) was the best method to obtain adequate material for sequencing [33].

The preparation of a library depends on the sequencing platform and the approach. However, in general terms, among the steps to generate the libraries, the fragmentation methods, the attachment of the adapters, and the quantification and library size determination should be considered. DNA fragmentation can be performed by physical, chemical, or enzymatic methods. Physical fragmentation is usually carried out by sonication, in which high-frequency acoustic energy is focused on the DNA sample to break up the molecules. In enzymatic fragmentation, the restriction endonucleases are the activities involved in fragmenting the DNA. An alternative enzymatic method for library preparation is tagmentation, which uses the transposase enzymatic activity to fragment DNA while adding specific adapters to both ends of the fragments (Illumina, San Diego, CA, USA). Therefore, it improves traditional preparation processes by combining DNA fragmentation, terminal repair, and adapter ligation in a single step, thus reducing the hands-on time. The attachment of adapters to the ends of the DNA molecules allows the identification of each processed sample. However, the existence of a high proportion of unattached adapters can cause adapter dimer problems. If these are not removed, they may result in a significant reduction in sequencing quality and efficiency. One of the most extended processes for their elimination consists of using magnetic bead-based clean-up steps. Regarding the fragment (also known as an insert) size, the optimal size is determined by the limitations of the NGS instrumentation and the specific sequencing application. With the current Illumina, Inc., technology, the optimal insert size is affected by the cluster generation process, where shorter products are amplified more efficiently than longer products. Therefore, assessing the fragment distribution of the final libraries is an essential QC step to ensure optimal results. This step could be automated using electrophoresis systems such as the TapeStation instrument (Agilent Technologies, Santa Clara, CA, USA). The evaluation of the quality of the final libraries is a critical step. Accurate quantification is essential since it provides an estimate of the molecules available to be sequenced in each sample. Quantification can be carried out using different methods, such as intercalating dyes, hydrolysis probes, droplet digital emulsion PCR, or fluorometry.

## 3. RNA Libraries

While this review has a focus on detecting and studying somatic and germline variation in melanoma, specific applications for assessing large structural variations that are important for cancer research could be based on transcriptomics, and some are discussed in Section 5.5. Because of that, we also provide some basics in case the starting material is RNA. For transcriptomic studies based on RNA-Seq, the typical steps include isolating the desired RNA molecules, reverse transcription to complementary DNA (cDNA), fragmentation or amplification of randomly primed cDNA molecules, and ligation of sequencing adapters [34]. The accuracy of gene expression quantification depends on the purity of the samples, and tumor tissue samples often comprise disease-state cells surrounded by normal cells. RNA library preparation also requires high-quality RNA isolated from the biological sample. RNA quality is commonly measured with a bioanalyzer (Agilent Technologies) or a TapeStation system, which provides an RNA Integrity Number (RIN) between 1 and 10, with 10 being the highest quality with minor degradation. Low RNA quality (RIN < 6) can strongly affect the sequencing results [35]. Alternatively, the quality of isolated RNA can be evaluated qualitatively based on the presence of intact ribosomal RNA (rRNA) bands on an agarose gel. As previously mentioned, FFPE tissues are usually of poor quality. Thus, the effect of RNA degradation must be carefully considered in the sequencing results [36]. Several commercially available solutions are well suited for FFPE and low-quality input samples.

The next step in RNA-Seq is library creation, which starts with the removal of remanent DNA and the isolation of the desired RNA molecules. There are several options in RNA-Seq library construction and experimental design to fit the specific needs of the researcher (poly-A selection, ribo-depletion, size selection, strand-specific, duplex-specific nuclease, multiplexed, short or long reads). In general, most RNA molecules in tissues are rRNA. Therefore, to detect less-abundant RNAs and for cost efficiency, it is necessary to remove rRNA transcripts before library construction. This rRNA depletion step avoids the consumption of the sequencing reads by rRNAs, increasing the overall depth of coverage of the RNAs of interest. Alternatively, messenger RNAs (mRNAs) are enriched by selection for polyadenylated (poly-A) RNA. The 3′ poly-A tail of mRNA molecules is targeted using poly-T oligos covalently attached to magnetic beads. Each methodological approach presents technical biases and limitations. Poly-A libraries are the best option for obtaining the coding RNA transcripts. While using rRNA depletion helps to accurately quantify non-coding RNAs and post-transcriptionally unmodified pre-mRNAs. Moreover, there are specific protocols for selectively targeting small RNA species, which are key regulators of gene expression. Small RNA species (15–30 nucleotides) lack poly-A and are microRNAs (miRNAs)—more than 800 miRNAs are deregulated in melanoma [37]—small interfering RNAs (siRNAs), and Piwi-interacting RNAs (piRNAs). Isolated RNA, with high quality and enough amount, is then fragmented, randomly primed, and subjected to the first and second cDNA strand synthesis. Finally, the adapters are ligated to the ends of cDNA fragments and amplified.

As in the case of DNA libraries, the protocol must add some QC steps. One consists of verifying the library profiles in an Agilent TapeStation system to ensure that their size is in the appropriate range and to determine the presence of unexpected peaks. The other QC is quantification, such as by quantitative PCR (qPCR), fluorometry, or the Agilent TapeStation system. The best method is qPCR since it quantifies the complete libraries, that is, those that can form clusters in the sequencing flow cell.

## 4. Sequencing-Based Approaches in Cancer and Cutaneous Melanoma Research

### 4.1. Sequencing with the Classic Approaches

The first generation, or Sanger sequencing [38], has been used to detect disease-causing variants [39,40,41], allowing the assessment of DNA fragments up to 1000 base pairs (bp) [42]. Being considered the gold standard in clinical research and key for assembling the first draft of the human genome for the Human Genome Project [43], the Sanger method has been used in melanoma research to characterize, for example, the particular behavior of it in different populations such as Taiwanese [44] and Chinese [45], among others. Some studies have shown a comparable performance between the first-generation sequencing with other variant detection approaches [46], with even some gains in efficiency and sensitivity in the case of the Sanger method [47].

In spite of the accuracy of the first-generation sequencing and the capability to evaluate repeated elements [48,49,50], the impressive development and throughput improvement in NGS have pushed aside the use of Sanger sequencing [42]. In this regard, pyrosequencing, the forerunner of NGS approaches, employs luminescence to identify nucleotides of the DNA strand based on the sequencing-by-synthesis (SBS) principle [51]. This sequencing technology has been used in melanoma research to unravel the clinical phenotypes related to *NRAS* and *BRAF* mutations [52,53]. Different commercial protocols have been developed to identify the most common mutations in codons within the *BRAF* gene, such as Therascreen™ BRAF Pyro Kit (Qiagen Inc., Valencia, CA, USA) for mutations in codons 464, 469, and 600. Additional molecular protocols for codons in *BRAF* (“BRAF Codon 600 Mutation Detection by Pyrosequencing”), *KRAS* (“KRAS Mutation Detection”), and *NRAS* (“NRAS Mutation Detection by Pyrosequencing”) have been conceived by ARUP Laboratories (Salt Lake City, UT, USA) to help in the treatment of patients with different solid tumors, including melanoma.

### 4.2. Next-Generation Sequencing

In 2005, a new sequencing system was released to the market based on pyrosequencing and emulsion PCR, allowing the parallelization of amplification reactions for the first time and a quantum leap in scale at the performance level [54]. This emerging technology, considered the first NGS system, opened the horizon for the development of many other approaches, which ultimately have resulted in an array of applications in clinical practice and biomedical research [55,56,57].

Since then, several others have emerged in this decade. Based on the sequencing chemistry, one can distinguish between sequencing-by-ligation (SBL), which uses a DNA ligase to add the nucleotides to the newly synthesized DNA molecule [58], and SBS, which uses a DNA polymerase instead of a ligase [59] (Table 1). An example of the first type is the SOLiD technology (Sequencing by Oligonucleotide Ligation and Detection) (Thermo Fisher Scientific, Waltham, MA, USA), whereas there are various commercial SBS-based sequencers, including Ion Torrent (Thermo Fisher Scientific, Waltham, MA, USA), MGI Tech (Shenzhen, China), or Illumina, Inc. (San Diego, CA, USA), among others. This review will focus on the applications based on the latter, since it is dominant in the market, because of its high versatility, performance, and market competitiveness. Despite this, it is worth mentioning that MGI Tech has become more and more popular in recent years because of its reduced costs and increased performance.

NGS allows to read billions of base pairs of DNA sequences quickly and simultaneously in only one experiment (“run”), resulting in a large dataset and an important cost efficiency. Routinely, an Illumina NGS experiment can be divided into the following four main steps: (1) fragmentation, (2) indexing or attachment of the adapters, (3) amplification, and (4) sequencing. As the two first steps have been extensively explained in the library preparation sections, we will now focus on the amplification and sequencing steps. One of the features of short-read approaches is the need for a PCR step prior to the sequencing run, which allows to establish the different clusters where the sequencing will take place. In Illumina instruments, classical amplification is produced by a bridge PCR. It means that one of the extremes of the single-stranded library attaches, by sequence complementarity, to one of the multiple single-stranded oligonucleotides on the coated surface of the flow cell. As this hybridization occurs, amplification begins immediately. A double-stranded molecule is provided, which is denatured, followed by a washout of the original template, whereas the covalent attachment of the newly synthesized strand is kept. This new molecule flips over and creates a bridge with a complementary oligonucleotide from the surface of the flow cell. Once a single-stranded library is bridged, the amplification starts and the library becomes double-stranded, coining this as the bridge amplification. Next, the denaturation step renders two single strands covalently bound to the flow cell. The bridge amplification continues until all oligonucleotides have been used. Afterward, linearization is carried out, the reverse strands are cleaved and washed away, and the forward strands are maintained on the surface. Finally, the 3′ extremes of the amplified fragments are blocked, and the sequencing primer is added to start the sequencing process. In the high-end of the throughput scale of Illumina (i.e., HiSeq 4000 and NovaSeq 6000 sequencing platforms currently) this process is also an exclusion amplification (ExAmp) to ensure that only one molecule attaching to each of the flow cell microwell forms a cluster. The patterned flow cells are also one of the exclusive features of the HiSeq 4000 and NovaSeq 6000 systems.

**Figure 1:**
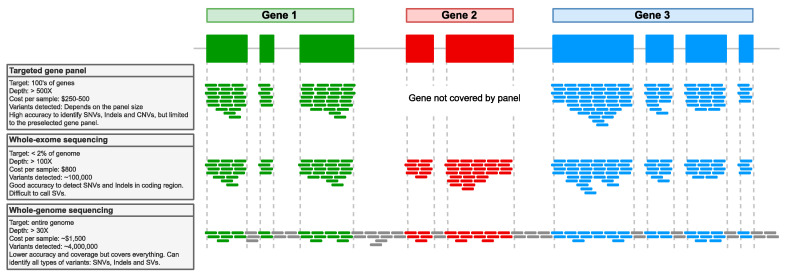
Overview of NGS DNA sequencing-based experiments applied on cancer genomics.

The NGS technology has been widely applied in cancer genomics, most commonly using short-read technologies and a high depth of coverage to study somatic variation. Based on that, the analysis of cancer samples, including melanoma subtypes [60,61,62], is typically performed using targeted sequencing of a cancer-specific gene panel, WES, or WGS (Figure 1).

Panel sequencing reduces costs, enables faster turnaround times, and requires a less complex pipeline for variant detection. Due to the remarkable benefits of this approach, different laboratories or manufacturers have developed their own panels to solve specific questions related to many types of cancers, such as the Hereditary Cancer Solutions by SOPHiA GENETICS (Boston, MA, USA), which allows assessing breast and ovarian cancer and some others involved in cancer-associated or predisposition genes in gastric [63] or pediatric cancer [64], among others. However, it has the disadvantage of not allowing the analysis of genes or genomic regions originally not included in the panel. Typically, these panels only cover driver mutations in genes known to be involved in melanoma, such as *BRAF*, *NRAS*, *KRAS*, *KIT*, *GNAQ*, and *GNA11* [65], and do not include genes that have been recently found by WES/WGS studies [16,66].

In a recent germline-focused study, the authors used WES and targeted gene panel sequencing of uveal melanoma samples, identifying associated susceptibility genes, and suggesting a locus heterogeneity in hereditary predisposition [62]. In another study, new therapeutic targets potentially related to alternative splicing caused by somatic mutations in multiple genes were specifically identified through WES [60]. Likewise, Vergara et al., using both WGS and WES data, analyzed the evolution of human melanoma from early to late-stage disease and found that it was dominated by tetraploidization and large-scale acquisition of aneuploidy [61].

WES enables assessing the mutational spectrum from virtually all the protein-coding regions, which harbor ~2% of the genome [67]. This cost-effective application allows the analysis of SNVs and small insertion-deletion variants (indels, around <50 bp in size) with high coverage reads. This implies that the data is more manageable, although of limited use to cover and identify larger structural variations (SVs) [68,69] that have key implications in melanoma [70,71]. Furthermore, familial pancreatic cancer [72], recurrent prostate cancer [73], malignant ovarian germ cell tumors [74], familial colorectal cancer [75], and locally recurrent rectal cancer [76] are just some of the cancers where the implementation of WES offers benefits by allowing the discovery of putative predictors and identifying risk genes and potential driver mutations involved in the pathogenesis. Whereas WGS allows to cover virtually all variation across the genome and to better unveil SVs. To date, several WGS studies have been carried out, focusing on the analysis of SVs in different subtypes of melanoma [61,66,77,78], but also to assess how mitochondrial genetic variation could influence gastric cancer [79], the discovery of novel mutations involved in prostate cancer [80], or even to study how the treatment could impact metastatic colorectal cancer [81]. In particular, the studies in melanoma have been able to demonstrate and identify key non-coding regions that are involved in the progression or risk of the disease and that cannot be detected with gene panels or WES.

## 5. Bioinformatic Workflows for NGS Data Analysis

Different advances and approaches in NGS are common in the toolbox of studies in cancer genomics and, specifically, in melanoma research [82,83,84,85]. NGS can generate different types of data, allowing to detect a wide variety of genomic abnormalities simultaneously. Besides, this technology can also help to analyze the molecular mechanisms of cancer, identify somatic mutations that have accumulated during tumorigenesis, and even assist in the discovery of new genomic, transcriptomic, and epigenomic profiles of individual malignant growths [82,83]. Likewise, the treatment of cancer patients could be improved based on NGS data, constituting one of the pillars of precision oncology [86,87].

In an effort to characterize cancer genomic alterations and their diversity, initiatives such as The Cancer Genome Atlas (TCGA) [88,89] and the International Cancer Genome Consortium (ICGC) [90] have gathered a vast number of cancer genomes from patient samples around the globe. In addition, the Genomics England 100,000 Genomes Project Cancer Program was established to develop a national molecular data research platform linked to longitudinal clinical data and to transform the National Health Service’s clinical cancer care based on WGS data [91,92]. Information collected as part of these studies includes clinical data, raw genomic data, and processed data. These data have been used not only for characterizing the mutational landscape of melanoma [93,94,95], but also to reveal mechanisms of tumor spreading [96], and identify biomarkers of treatment response [97], among many others.

A common method is to analyze paired normal and cancer tissues from the same patient and use the normal as a comparator [98]. As sequencing costs continue to decline, sequencing platforms are being redesigned to prioritize WGS variant reporting based on clinical relevance [99]. Thus, current trends are focusing on developing new bioinformatic algorithms for both WES and WGS in order to improve their clinical application [18,100]. Interestingly, developments and improvements in algorithms in the context of cancer genomic data analysis make it possible to provide a probability score of disease-driving mutations and to identify other potential targets [101,102].

Given the large amount of data that is generated as part of the NGS experiments, managing, administering, and storing such large sequence datasets, as well as the need for an efficient analysis, is a real challenge. Storage requirements for raw, intermediate, and processed data critically depend on the type of experiment as well as a number of parameters, such as the depth of coverage or the number of different variant detection tools that will be involved. As expected, experiments based on tumor-normal pairs or on WGS need a larger amount of disk space. Bioinformatic analysis typically begins with the raw data sequencing and ends with a listing of somatic variants per sample or aggregate across the samples of the experiment. These steps include processing raw reads, alignment to the reference genome, variant calling, annotation, filtering, and prioritization of variants. However, the software tools and workflows to be used depend on the type of experiment and the processing strategy applied. Currently, there is no single gold standard processing strategy for cancer data, and each pipeline implements these steps, or most of them, using different tools and parameters. Some of the state-of-the-art workflows for cancer genomics are the NYGC Cancer Pipeline [103], Sarek [104], and the GATK Best Practices for both somatic short and copy number variant (CNVs) discovery [105].

**Figure 2:**
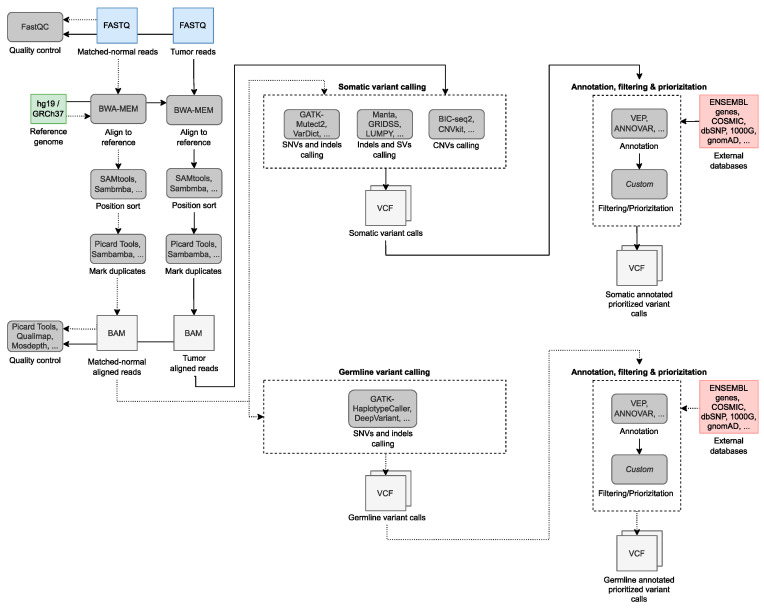
Schematic overview of the pipeline for WGS somatic mutation identification.

Moreover, the process of aligning reads with the reference human genome, variant calling, and assembly for cancer WGS data requires a large amount of computational power at each analysis step (Figure 2) [106]. In this context, for faster data throughput as well as to significantly simplify and reduce disk space requirements, it is key to focus on combining as many of these steps or computational tools as possible using Unix pipes. In this way, for workflow standardization and automation, several managers are available, such as Snakemake [107], WDL [108], or Nextflow [109]. To overcome the limitations of hardware and support required for large-scale genomics projects, there are high-performance computing (HPC) facilities. These are equipped with a cluster of high-speed computing nodes and multi-petabyte storage systems, enabling distributed and parallel computing, cloud computing, and graphics processing unit (GPU) computing, among others.

From here on, each step of a typical pipeline for cancer genomics will be briefly described, indicating the most common tools and peculiarities for different types of experiments.

### 5.1. Read Alignment to the Reference Genome

Paired-end read sequence data is generally provided as two files in FASTQ format, each file representing one end of the read. The sequence data is stored in the FASTQ files as plain text and contains the sequence of the read and the per-base quality scores. In a typical pipeline, the sequence files are aligned to the reference sequence using an aligner. Different builds of the human reference genome are available, with GRCh37 (hg19) and GRCh38 (hg38) being the most popular. In practice, hg19 is still the most widely adopted, as most vendors provide their probesets in hg19 coordinates [110]. Recently, the Telomere-to-Telomere Consortium (T2T) has generated a gapless reference of the human reference genome [111]. Besides, the T2T-CHM13 assembly corrects and expands the sequence coverage of the GRCh38 human genome by more than 200 Mbp, including highly repetitive DNA sequences at telomeres and centromeres of the 22 autosomes and the X and Y chromosomes [111]. This new assembly has enabled many previously unknown genes to be identified and has been shown to significantly reduce false positives in hundreds of medically relevant genes [112]. A wider adoption of this new assembly, including in clinical practice, will depend on creating new annotations and a liftover of the existing major genome annotations to T2T-CHM13 [113].

To perform short-read alignment of gene panel, WES, or WGS data, BWA-MEM [114] is one of the most widely used aligners. Other popular alignment tools include Novoalign [115], Bowtie2 [116], or Minimap2 [117]. The resulting aligned sequences and their related metadata are stored in the SAM/BAM file format (Sequence Alignment Mapping). This file is subsequently sorted by genomic coordinates and indexed for quick access. SAMtools [118] is the most used tool to manage SAM/BAM files since it allows us to carry out most of the operations. Marking or removing read duplicates in the BAM file is a crucial step to account for PCR duplicates of the exact same DNA fragment and limit their impact in the variant calling stage. Tools such as Sambamba [119] and Picard [120] are commonly used to identify and mark duplicate reads in SAM/BAM files to exclude them from subsequent analyses. Downstream analyses rely on the SAM/BAM files to identify a wide range of genetic variations. QC steps of SAM/BAM files should be made prior to variant calling to evaluate the sequencing metrics, assess the depth of coverage and the percentage of duplicate reads, evaluate sample contamination, or perform sex inference.

Depth of coverage is a key metric to evaluate, often defined as the average number of non-duplicated reads that align across the target region. The target region can be the exons of a gene panel, the targeted exons across all genes, or even the entire genome. The level of coverage often determines whether variant discovery can be performed with a certain degree of confidence at a given genomic position. Typically, the coverage needed for an NGS experiment is determined by the method being used and the characteristics of the experiment. This metric should be calculated on both normal and tumor BAM files and can be easily obtained using tools such as Mosdepth [121]. For gene panel and WES data, there are also a number of key parameters to consider, as indicated elsewhere [67]. On-target mapped reads and on-target coverage should be calculated to assess potential problems during the library preparation. Picard Tools and Qualimap [122] are usually used for this purpose. MultiQC [123] can also be helpful to aggregate QC results from different bioinformatic tools and different experiments into a single report.

### 5.2. Variant Calling of SNVs and Indels

The alignment results in BAM format are subsequently examined for the presence of any type of somatic variation. The accurate identification of mutations is of critical importance. Numerous variant callers are available for this purpose. A list of the most widely used SNV and indel callers can be found in Table 2. To distinguish germline from somatic mutations in the tumor, a common practice is to rely on a normal tissue sample from the same individual. Somatic callers such as GATK-Mutect2 [124], Strelka2 [125], and VarScan2 [126] consider simultaneously the aligned data from the tumor and normal samples.

Sequencing the tumor and normal sample genomes allows not only the identification of variants with greater fidelity but also allow the finding the of potential therapies (see references in Table 2). If sequencing data from the normally matched sample is available, it is also recommended to run a germline variant calling to detect variants that may indicate possible susceptibility to cancer or may be useful in treatment responses. GATK HaplotypeCaller [105] and DeepVariant [127] are widely used tools for this purpose.

**Table 2:** Most common tools to call somatic variants and related research studies in cancer genomics.

Somatic Callers	Sequencing Approach			Type Mutations	Normal Sample Required in Somatic Mode	Related Somatic Studies
	Targeted	WES	WGS			
GATK-Mutect2 [105]	✓	✓	✓	SNVs and indels	Optional	Liver cancer [128], lung cancer [129]
Strelka2 [125]	✓	✓	✓	SNVs and indels	Yes	Cervical cancer [130]
VarDict [131]	✓	✓	✓	SNVs and indels	Optional	Breast and ovarian cancer [132]
CNVKit [133]	✓	✓	✓	CNVs	No	Melanoma [134]
Manta [135]	✓	✓	✓	SNVs and indels	Optional	Gastric cancer [136]
Delly [137]	x	x	✓	SVs	Yes	Plantar melanoma [138]
Lumpy [139]	x	x	✓	SVs	Optional	Colon cancer [140]
GRIDSS [141]	✓	✓	✓	SVs	Yes	Myeloid leukemia [142]
Varscan2 [126]	x	✓	x	SNVs and indels	Yes	Uveal melanoma [143]
ClinCNV [144]	✓	✓	✓	CNVs	Yes	Cutaneous leukemia [145]
ExomeDepth [146]	✓	✓	x	CNVs	No	Breast cancer [147]
ClinSV [148]	x	x	✓	SVs	No	Breast cancer [149]

WES, whole-exome sequencing; WGS, whole-genome sequencing; SNVs, small nucleotide variants; indels, insertion-deletion variants; CNVs, copy number variants; SVs, structural variants.

As an important remark, some somatic variant calling tools require the normal matched sample, conditioning the choice of the variant caller. When the normal matched sample is not available or useful (i.e., due to technical reasons), several tools allow the use of a “panel of normals” (PoN), made out of sequencing data from normal unrelated individuals (N ~50). The PoN can be used to filter out variant calls associated with recurrent technical artifacts, systematic noisy positions, and germline variants, although its effect is limited since this approach does not eliminate the germline variants of the individual.

The performance of somatic variation detectors varies widely, as demonstrated in various benchmarking studies, each showing strengths and weaknesses [150,151]. The precision of the detection depends mainly on the sequencing depth in each genomic region and on the alignment or mapping error. Considering the complexity of the human genome, especially in non-coding regions, mapping short reads to repetitive regions and tandem repeats typically imposes difficulties, resulting in reduced sensitivity and specificity of most variant detection tools. Because no somatic variation detector has yet surfaced as the gold standard because of a superior performance across all scenarios, a joint approach that combines the results of two or more complementary callers provides a better balance between sensitivity and specificity [150].

Indels have an important role in tumorigenesis, especially if they affect the coding region, where they can significantly disrupt the reading frame and lead to changes in protein function. Because indels have not been studied as thoroughly as SNVs, tools, and methods for indel detection typically need to be fine-tuned and optimized. In this context, initiatives such as NCTR Indel Calling from Oncopanel Sequencing Challenge (https://precision.fda.gov/challenges/22, accessed on 3 September 2022) aim to improve indel detection by validating and benchmarking indel calling pipelines across laboratories.

After the variant calling step, the resulting variant callset is typically reported in variant call format (VCF), encoding metadata and variant records for each sample. VCF files are often compressed and indexed so that they take up less disk space and can be handled more efficiently by applications. Widely used tools for managing VCF files are BCFtools [118] and VCFtools [152].

### 5.3. Variant Calling of SVs and CNVs

Structural alterations, including large insertions and deletions, duplications, inversions, translocations of at least 50 bp in size, and gene fusions, have been associated with cancer pathogenesis. Large deletions and amplifications, occasionally spanning genes, or even entire chromosomes, sometimes lead to alterations in gene copy number. This type of SV is usually referred to as CNVs and copy number aberrations (CNA). In most cancer types, including melanoma, a remarkable number of somatic CNAs accumulate during the progression of the disease and have been associated with cancer prognosis and development. CNAs have been directly associated with the expression of driver genes, where copy number changes may increase the expression of oncogenes and decrease the expression of tumor suppressor genes [153,154,155].

For SV calling based on NGS paired-reads data, SV detection tools typically rely on one or a combination of the following approaches: (a) coverage depth (RD), in which changes in coverage may imply an SV, (b) discordant read pairs (RP) in the alignment, where read pairs map at unexpected distances or orientations, (c) split-read mapping (SR), in which part of the read aligns to either side of an SV, (d) and the assembly approach (AS), which detects SVs by assembly-based sequence reconstruction. The best-performing detection tools usually leverage a combination of some of the above methods [156].

Popular tools for SV detection include Manta [135], DELLY [137], LUMPY [139], GRIDSS [141], and CNVKit [133] for WGS data (see Table 2), some of them being adaptations from germline CNV/SV calling. Many of these tools have been benchmarked and ranked in review studies, providing mixed conclusions [157,158].

In targeted sequencing of a gene panel and WES, only the approach based on the variation in depth of coverage (RD) can be applied; hence, only CNVs can be reliably detected in such experiments. The sparse distribution and small size of exon targets make the relationship between copy number and depth of coverage more complex, making CNV detection less successful. This discontinuous data is skewed by technical limitations arising from GC content bias, non-uniform sequencing depth, and PCR amplification artifacts. In order to mitigate some of these limitations, CNV callers for target sequencing/WES usually perform multi-sample normalization and implement several model-based approaches by using samples sequenced on the same equipment and with the same sequencing kit for better results. Some of the most used CNV detection tools in these experiments are ClinCNV [144], CNVKit [133], and ExomeDepth [146].

Similar to somatic SNV and indel calling, combining the results of at least two of the tools based on different approaches results in an optimal strategy for somatic SV/CNV calling [159]. In the detection of somatic SVs, a matched normal sample is also usually required to be used as a comparator.

Although the detection of SNVs and indels based on NGS data can be considered routine, the detection of SVs even with WGS data still poses many challenges. This is mainly because a large fraction of SVs are found in difficult-to-map regions of the genome, such as repetitive regions or tandem duplications, which impose uncertainty during the aligning process. Additionally, short reads are often insufficient to resolve complex SVs and long insertions, as these can be smaller than the SV sizes. All these may result in miscalling events or provide false positive and false negative calls. For this reason, linked reads and long-read-based sequencing are increasingly being applied to the detection of SVs to achieve higher levels of sensitivity and specificity in the studies.

This is an active area of interest but is still unresolved. Initiatives such as the precisionFDA challenges are aimed to benchmark the state-of-the-art variant callers in challenging genomic regions, especially those important for medical sequencing [160,161].

### 5.4. Variant Annotation, Filtering, and Prioritization

After variant calling, the identified variant callset, including SNVs, indels, CNVs, and SVs, needs to be annotated with functional information to assess the biological implications. The accurate identification of somatic variants is essential to provide potential candidates to be used in targeted cancer therapy [162]. This process includes annotation to identify if the variant affects the protein coding sequence of a gene, splicing, and other regions, as well as pathogenicity scoring and effect prediction of being a carrier of the variant. Additionally, the variant callset is typically annotated with existing population information from databases and studies, such as the NCBI dbSNP [163], gnomAD genome/exome [164] frequency data, etc., to identify if a variant has been previously identified by another study of germinal variation or COSMIC [165] to assess if it has been previously associated with any type of cancer. Commonly used variant annotation tools such as Ensembl Variant Effect Predictor (VEP) [166], ANNOVAR [167], or GATK Funcotator [168] annotate variants individually in the VCF file. Depending on the reference transcript used by the tool, typically RefSeq or Ensembl, the functional annotation may vary [169]. Hence, the choice of the annotation tool must be performed carefully.

After the annotation step, the variant calls should be filtered to remove common alignment artifacts and reduce the number of false-positive somatic calls. The annotation information is very useful because it enables the filtering the variant callset. Population filtering is also a common strategy for identifying and filtering likely germline variants from somatic mutation callsets. However, this step must be performed carefully, as common databases such as dbSNP and gnomAD contain several mutations from human tumors, whereas somatic variant catalogs, such as COSMIC, contain germline variants. Similar to SNV and indel calling, CNVs and SVs can be filtered against a PoN to remove variants in highly variable regions and artifacts. Germline SV databases such as gnomAD-SV can also be used to filter SVs that are variable in otherwise healthy human populations.

A manual review of tumor and normal sequencing alignments using visualization tools such as IGV [170] can help in eliminating false positive somatic calls. A manual review of CNVs and SVs can also be performed in the alignment file. This may be useful to resolve ambiguous SV breakpoints, although sometimes the variation occurring is difficult to deduce. SVs with well bioinformatic support are often supported by both discordant read pairs and changes in sequence coverage in specific regions. Tools such as Samplot [171] enable the identification of false positive SV calls using visualizations, whereas Samplot-ML [171] is able to discriminate between true and false deletions using convolutional neural networks (CNN) for image recognition [172].

Besides manual review, a subset of the detected variants can be independently validated by orthogonal approaches, such as Sanger sequencing. Despite the filtering protocols that can be implemented, most NGS methods detect many more candidate variants with likely functional effects than it is possible to validate experimentally as part of a project. Variant prioritization is a common practice to obtain a manageable set of variants. Although variants can be ranked based on various parameters, the prioritization of candidate variants that may be related to the disease is a multifactorial problem, and generally represents a bottleneck in cancer genomics. Open access databases such as the Clinical Interpretation of Variants in Cancer (CIViC) [173] have accumulated and curated information from diverse cancer types and are useful to identify cancer biomarkers and variants that may be used for treatment response. Typically, this task has been performed by experts in the biomedical field. Unsupervised and semi-automated techniques have emerged in recent years [174,175], although none of them has become the gold standard.

### 5.5. Tumor Clone Identification

High-throughput sequencing enhances the study of tumor evolutionary patterns, enabling the deciphering of all mutations in tumor clones [176]. Tumor clones are clusters of cells that share several somatic mutations, and the evolution of each clone can be represented by the variant allele frequency (VAF) [177]. VAF can be obtained by NGS and is defined as the percentage of reads that match a specific variant divided by the total coverage at that variant locus [178].

Assuming that almost all somatic variants in tumor cells are heterozygous, the proportion of tumor cells with the same mutation is twice the VAF value. This means that a specific variant present in 80% of tumor cells will have a VAF value of 40%. Using a VAF density plot, tumor clones and the present variants in each clone can be represented by each peak in the plot, helping us to infer and identify clonal progression [179]. Initially, in tumor population cells the VAF of the existent mutations would follow a normal distribution with a value near 50%. This means that a clone cell that carries a mutation set is also present in almost all tumor cells. With the clonal evolution of the tumor cells, new mutations may emerge, causing one of the new clones to carry both the original and the new set of variants that may provide survival advantages to the cell. In this case, if half of the tumor cells belong to the new clone, the VAF density plot would have two peaks at 50% and 25% VAF. With the following tumor progression, some clonal cells obtain a third set of mutations, producing more potentially malignant cells, which can lead to adverse effects such as metastasis. Assuming that a quarter of the tumor cells have the third set of variants, the VAF density plot would have three peaks at 50%, 25%, and 12.5%.

### 5.6. Gene Fusions

**Figure 3:**
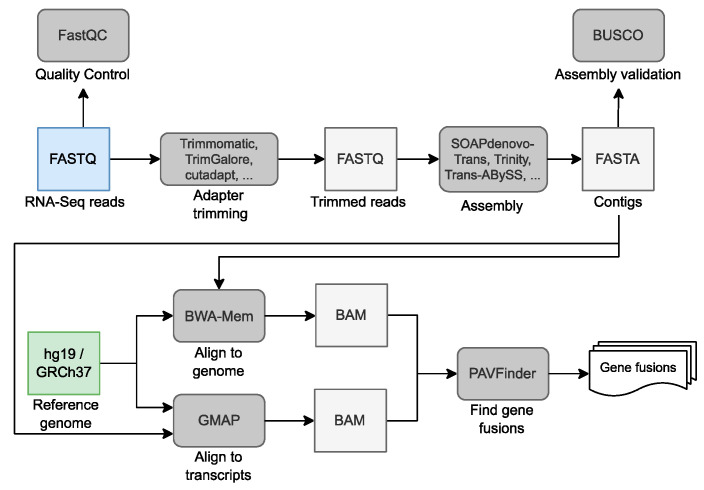
Bioinformatic pipeline for gene fusion discovery through RNA-Seq data. Reads are subjected to a trimming process by means of tools such as Trimmomatic [184], cutadapt [185], or TrimGalore [186] in order to remove adapter sequences in the data. Then, trimmed reads are used for the de novo assembly process, producing assembled contigs in FASTA format. SOAPdenovo-Trans [187], Trinity [188], or Trans-ABySS [189] are examples of RNA-Seq assemblers. These contigs are aligned both to the reference genome and to the reference transcripts. Finally, resulting alignment files in BAM format were analyzed using PAVFinder [190] in order to discover transcriptomic SVs as gene fusions or tandem repeats.

Gene fusions (also known as chimeric transcripts) can be caused by somatic chromosomal rearrangements involving large SVs or chromosomal translocations. Gene fusions have been involved in the progression of a variety of cancers [180], including melanoma [181], lung cancer [182], and breast cancer [183]. These mutations can also be generated at the RNA level by the co-transcription of neighboring genes or by splicing processes from different genes. As such, these rearrangements may be more efficiently associated with NGS-based transcriptomics. In this context, transcriptomic analysis using RNA-Seq data has emerged as a promising solution to identify gene fusions of potential importance for cancer development. A simple computational workflow for accurate detection and characterization of fusion transcripts from RNA-Seq, including alignment and variant analysis steps, is presented in Figure 3.

The most common way to shed some light on cancer-related gene fusions is to run de novo assembly and annotation of transcripts using short reads from RNA-Seq [191]. To carry out this process, a variety of transcriptome assemblers [189,192], SV callers, and full pipelines [193] have been developed in recent years. There are also tools such as INTEGRATE [194] that combine WGS and RNA-Seq data from the same sample to discover expressed gene fusions in cancer cells. Methods that are not based on transcriptomes but on de novo assembly of WGS long-read data are also starting to emerge as an alternative way to deeply assess somatic SVs (including gene fusions) in cancer genomes. Further details are provided in Section 6 of this review.

### 5.7. Further Quality Control Steps to Perform in the Callset

The massive amount of data generated by NGS technologies need to establish standardized procedures, guidelines, and rigorous QC steps to ensure accurate results. More importantly, these steps are key for the implementation of NGS technologies in clinical areas, where high-quality data and reliable results are fundamental [195]. Many QC steps are applied throughout the bioinformatic workflow. Different tools, such as FastQC [196] and Qualimap 2 [122], perform the QC based on raw reads and read mapping, respectively. However, these have been covered in previous sections. As important as these are the traceability of samples to verify which results correspond to which samples when processing multiple samples in parallel. For this purpose, studying family relationships within a cohort or inferring sample sex are essential steps to help sample tracking.

#### 5.7.1. Relatedness

Traditionally, pedigrees were the gold standard to infer relatedness, but the advent of new technologies and the availability of different genetic markers make it possible to evaluate relatedness using many approaches. Tools to infer relatedness exist for the SNP array technologies, such as KING [197], REAP [198], or KIND [199], which could be used in this context. Specifically, for NGS data, tools such as Somalier [200] are very convenient to infer family relationships with data from diverse applications (WGS, WES, RNA-Seq, etc.). Relatedness among samples is calculated by allelic concordance from SNVs within these positions (classified as homozygous, heterozygous, and alternative homozygous).

#### 5.7.2. Sex Inference

**Figure 4:**
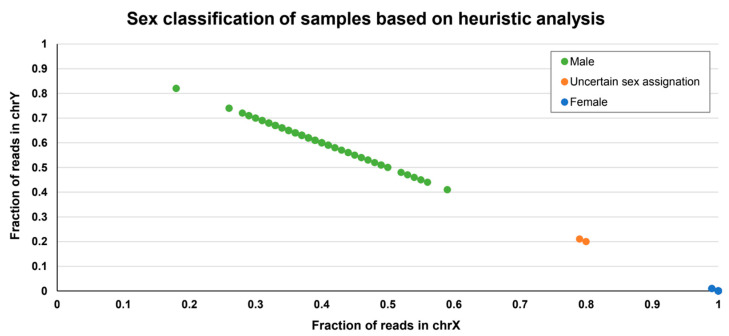
Typical sex inference of multiple samples based on read depth of X and Y chromosomes heuristic analysis. Uncertain sex assignation can aid the detection of sequencing errors in a multi-sample project [202].

The genetic inference of the sex of a sample from its sequence obtained in an NGS experiment is a mandatory QC step to detect errors in the metadata, which helps to improve sample traceability. Several bioinformatic tools are available to infer the biological sex of the sample based on the proportion of reads aligning to X and Y chromosomes, such as XYalign [201] (Figure 4). Some others are based on the depth of coverage of the X and Y chromosome reads at selected genomic positions, such as that conducted by Somalier [200].

## 6. Long-Read Sequencing Technologies in Cancer Genomics

A broad range of long-read sequencing technologies, also known as TGS, is flourishing and being used to improve our knowledge of complex regions and SVs that were difficult to resolve or were missed by short-read sequencing analysis [203]. Long-read sequencing is quickly evolving and becoming prevalent in cancer studies, allowing us to fully characterize novel somatic mutations involved in cancer, such as CNVs and SVs [204], and helping to identify further driver genes.

Several long-read sequencing technologies have been developed recently (Table 3). However, the prevailing TGS technologies are now the ones developed and marketed by ONT and PacBio. Other approaches, such as linked-reads [205], chromosome conformation capture sequencing (Hi-C) [206], and optical-mapping [207], are worth highlighting as well since they have demonstrated utility in cancer research.

ONT has marketed a number of platforms such as the MinION, GridION, or the PromethION, among others, which are capable of sequencing ultra-long fragments of DNA or directly of RNA, offering also real-time analysis [214]. The MinION, released to the market in 2014 and the first device using nanopore technology, is a portable sequencer capable of sequencing whole small genomes or exomes, metagenomes, and transcriptomes [215,216]. GridION runs up to five parallel MinION flow cells and is suitable for medium-scale projects such as larger genomes, whole transcriptomes, or large numbers of samples. PromethION has 24 or 48 parallel flow cells and is a high-throughput device for large-scale projects, suitable for larger genomes and population sequencing. Lower-scale variations of PromethION are about to be released. These devices use flow cells that contain an array of multiple and parallel nanopores embedded into an electrically resistant polymer membrane. Single-stranded DNA or RNA molecules can pass through these nanopores with the help of proteins by means of an ionic current produced by applying a constant voltage. As nucleotides pass through, the current is disrupted, producing a characteristic change [217]. This signal is measured and then decoded using basecalling algorithms to determine the corresponding nucleotide type in real-time [218]. Theoretically, the length of the reads has no limits. However, in practice, the longest reads are currently capped at a maximum size of ~2 Mb imposed by the challenges of handling very long DNA molecules.

PacBio uses proprietary SMRT (single-molecule real-time) technology [219], a method based on a single-DNA polymerase attached in zero-mode waveguides (ZMWs), subwavelength optical nanostructures, to detect fluorescent signals. PacBio released the Sequel system in 2015, a platform that uses this sequencing process to obtain long-read sequences longer than 10 kb. In early 2019, PacBio released the Sequel II sequencing platform, an improvement of the Sequel I platform with higher data output. Moreover, together with Sequel II, they developed circular consensus sequencing (CCS), an improved method to produce HiFi (high-fidelity) sequences with high base accuracy (>99%) in reads of about 10–15 kb in length. This makes PacBio technology suitable for de novo assembly, RNA sequencing, or comprehensive variant detection [220].

Another approach, known as linked-read technology [221], was developed by 10X Genomics and had a significant impact on the analysis in the determination of phased haplotypes and the identification of large genomic rearrangements. However, this sequencing method has been discontinued. New alternatives, such as the Hi-C technique [222], a method developed to study 3D genome folding, generate synthetic long-reads using short-reads while adding information from long DNA strands [223]. This methodology helps to build chromosome and genome structures, improving phasing and scaffolding to provide high-quality draft assemblies [224], helping to explain events such as genome folding or gene regulation [225]. It is also worth noting the utility of optical mapping technologies such as BioNano, which labels DNA sequences and then generates genome maps, to improve the assembly by scaffolding the assembled contigs [226] or to discover large SVs [227,228].

In 2020, the T2T announced the first gapless de novo assembly of a human X chromosome using ultra-long DNA reads from ONT sequencing of the T2T-CHM13 genome [229]. In 2022, combining several of these technologies, the T2T reported the first complete human genome, unraveling the last 8% of the genome that remained unresolved [111]. Despite there is yet a lack of melanoma studies leveraging these technologies [95], these will bring improvements to the field, by allowing the implementation of new reference databases, reducing variant calling errors, and improving genetic analyses of de novo and somatic mutations [112].

### 6.1. Advantages and Limitations of Long-Read Sequencing in Cancer Genomics

Long-read sequencing offers several advantages over short-read sequencing approaches. They are particularly useful in de novo assembly strategies because, by solving the low complexity and repetitive regions, they can reconstruct accurate and high-resolution genome assemblies [230]. This also has a positive effect on the ability to resolve repetitive and difficult to map regions [160], the comprehensive discovery of large and complex SVs, the full characterization of transcriptomes and the alternative splicing transcript species, the capacity for variant phasing in chromosomes, and the direct detection of epigenetic changes [231]. In the case of ONT, the MinION sequencing device allows to sequence samples in real-time and in a portable way [215]. All these benefits make this technology very promising for the characterization of any cancer type, as well as for developing specific therapeutic strategies [208].

As previously mentioned, TGS has several technical advantages and improvements compared to the traditional NGS. However, there still exist some limitations and challenges that are associated with these technologies, which may explain why their use in cancer research is not that widespread yet, particularly in the case of melanoma [95]. The main disadvantage of this sequencing technology is its higher error rate, which translates into a lower accuracy in the subsequent analysis. Nevertheless, base calling and error correction algorithms are continuously improving and progressively allowing to obtain improved read accuracies, which have a strong impact on read alignment and variant detection [232]. In addition, sample requirements for sequencing long-reads are substantially higher compared to short-read technologies. The amount of input DNA required nowadays is particularly problematic in tumor samples, as the starting material of these samples is usually limited and is typically degraded if the source is FFPE samples. In order to maximize the yield from sequencing and improve the quality of the data obtained, specific protocols for DNA extraction are necessary [233].

### 6.2. An exemplar Application of WGS with Long Reads from ONT

As discussed before, long-read sequencing platforms offer advantages for the de novo assembly of genomes compared to the classical NGS methods [234]. ONT and PacBio have accelerated computing times and increased the read length up to several thousand base pairs, despite the higher error rate, making it possible to assess human genome variation from de novo assemblies [235]. Longer sequences make it easy to find overlaps with other sequences of the experiment, obtaining better results by correctly assembling DNA fragments and facilitating the spanning over repetitive genomics regions (as gene duplications, transposons, or satellites). Because of these improvements, it is possible to run de novo assembly to obtain a personalized genome and use it as a reference for detecting somatic events from cancers [236]. Using a personalized genome assembly [237], rather than the standard reference such as GRCh37 or GRCh38, as a reference for tumor samples could improve read alignment and somatic mutation discovery.

#### 6.2.1. Library Preparation and Sequencing

ONT provides a comprehensive range of DNA and RNA library preparation kits, offering high throughput with low DNA input, fast modes for library preparation, and long reads. This technology has a wide variety of solutions, including WGS, targeted sequencing, and RNA sequencing, among many others. Whereas with other sequencing technologies the read length is limited by the technology itself, in the case of long reads this limitation is given by the quality and size of the starting DNA material. Thus, the challenge is in how to improve DNA extraction to preserve DNA purity and integrity. For analyzing human cancer WGS, one can proceed with the ONT ligation-based library preparation kit (SQK-LSK110) and a PromethION platform, which can generate up to 150 Gb of sequence per flow cell and offers 30× coverage WGS for less than 1000 dollars [238].

**Figure 5:**
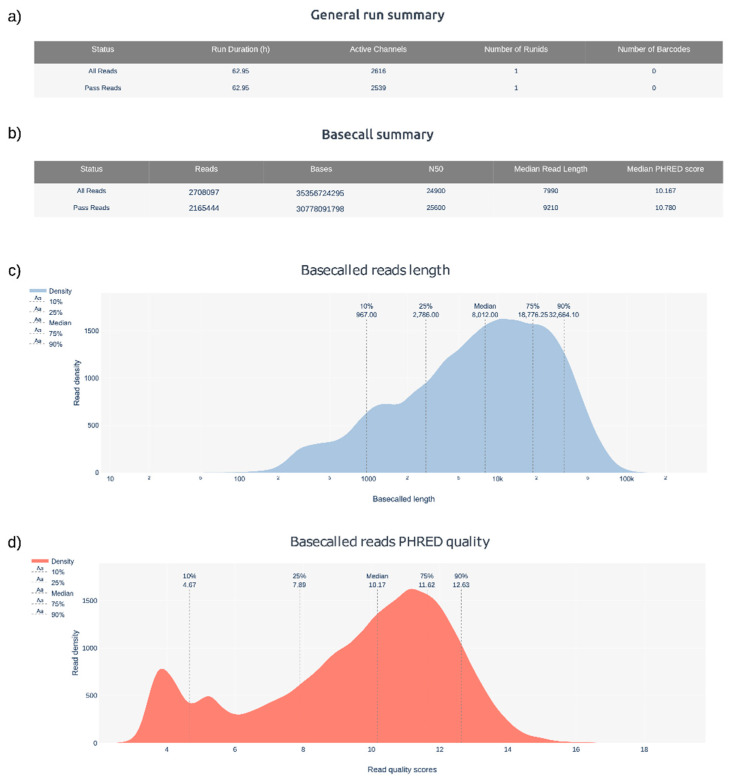
Summary report of ONT basecalling generated by means of pycoQC [239]. (**a**) General run statistics; (**b**) basecalling summary: number of total reads and bases, N50, median read length and median score; (**c**) plot of basecalled reads length; (**d**) plot of basecalled reads Phred scores.

Signals from the sequencing process are stored within FAST5 format files and could be processed by basecalling algorithms, such as Guppy or Bonito (ONT), to decode the sequence into FASTQ files. Basecalled reads can be inspected using tools such as pycoQC [239] (Figure 5) or NanoPlot [240] to generate interactive QC metrics and plots.

#### 6.2.2. Bioinformatic Tools for Long-Read Analysis

In recent years, the research activity involving long-read technologies has grown rapidly [238,241]. This effect produces an exponential development of bioinformatic tools for long-read sequence analysis. Some of those tools are maintained by companies in their own repositories, while others are open-source applications and pipelines developed by researchers or laboratories. Table 4 provides some of the most common computational tools and applications for long-read sequencing data analysis, including basecalling, error correction, and de novo assembly, among others.

#### 6.2.3. De Novo Genome Assembly

**Figure 6:**
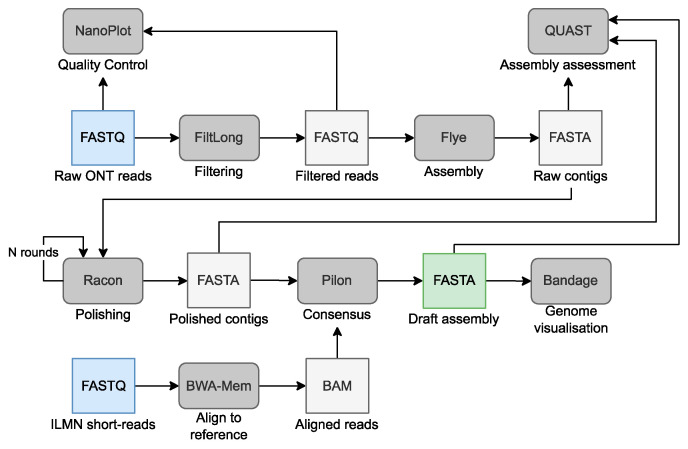
Basic de novo genome assembly pipeline using long reads from ONT. This pipeline starts by filtering raw ONT long reads using FiltLong to discard short and low-quality reads. NanoPlot is then used to check the quality of both raw and filtered reads. The remaining reads are de novo assembled by using Flye. Resulting contigs from assembly are polished with multiple rounds of Racon and Pilon, in case that short reads of the same sample are available. Resulting assemblies can be assessed using QUAST and visualized with Bandage.

De novo assembly of genomes is one of the main uses of the long reads generated by ONT and PacBio sequencing. The main goal of de novo assembly is to attempt to reconstruct the whole genome sequence without information from a reference genome [224,262] or in situations that aim to avoid potential biases from using the standard reference genome [237,263,264] that could be incomplete or not be representative of the population of the study [226,265,266]. De novo assembly algorithms manage to build contiguous and accurate sequences that represent the genome of the analyzed individual without information on the structure and complexity of the genome. There are two main algorithmic strategies to run de novo assembly, one based on building a de Bruijn graph (DBG) and the other based on the overlap layout consensus (OLC) algorithm [267,268]. A basic workflow to complete a de novo genome assembly using Flye [253] as the assembler and using both long and short reads to run several polishing steps is shown in Figure 6.

The quality of the assemblies could be improved by combining data from different sequencing technologies, such as long reads from ONT and short reads from Illumina, Inc. With this approach, tools such as MaSuRCA [257] run hybrid de novo assemblies, using the long reads to scaffold contigs generated by short reads to solve regions that cannot be resolved using short reads alone [269].

#### 6.2.4. SV Calling

Identification of small variants (SNVs and indels <50 bp) is essentially well resolved with short reads. However, the difficulty increases when it comes to detecting larger sequence mutations with this technology [157], and it is especially complex when dealing with somatic variants due to the purity and heterogeneity of tumor samples. These limitations have hindered the study of SVs in the past, despite their interest in cancer. Nowadays, powered by TGS technologies and the ability of long reads to cover repetitive elements and large and complex regions, the detection of SVs is more manageable by calling tools [270] and allows a more precise characterization of the SVs of the human genome [271]. Nevertheless, two of the previously mentioned disadvantages must be considered for alignment and SV calling the following steps: the high error rate and the throughput (translated into lower coverage) compared to short-read sequencing.

SV methods with long reads are mainly focused on the following two strategies: one based on aligning reads to a reference and another based on de novo assembly. Approaches that use de novo assembly are based on assembling reads to longer sequences, namely, contigs or scaffolds, and discovering SVs by comparing aligned reads to the assembled sequences and to a reference [251]. On the contrary, read alignment approaches are based on aligning raw reads against a reference and analyzing the resulting alignments to detect SVs.

A large number of tools rely on these methods, and their comparison helps in the selection of the optimal tool and the strategy for SV calling [272]. The application of long-read sequencing technologies to study SVs has increased recently in cancer studies [19,273], although there are still very limited examples of this in melanoma research [274].

## 7. Discussion

NGS platforms have made a huge impact in the characterization of human tumors, helping to understand and identify several types of cancer and establish new targeted treatments. Moreover, despite the technical challenges that are due to the quality and quantity of the tumor samples, advances in sample preparation methods have enabled the full characterization of cancer genomes, transcriptomes, and epigenomes [275]. These advances are allowing the improvement our understanding of cancer-specific small-sequence mutations, such as SNVs and indels, and large genetic variations, such as CNVs or structural rearrangements.

Bioinformatics pipelines for the analysis and clinical interpretation of cancer genomic results have been implemented in many platforms and laboratories. Likewise, multiple computational tools have been developed for the analysis of oncological NGS data. Most of these tools are used through the command line, sometimes being complex to parameterize and optimize. The choice and application of these tools depend on the characteristics of each specific project. Thus, the optimal configuration must be determined empirically for the sequencing strategy, the sample type, and the computational resources.

Advances in NGS technologies, improvements in the variant detection algorithms, and further development of specific human cancer databases and functional annotations of the genetic variants, as well as the reduction of the cost of sequencing, will impact the field of cancer genomics, bolstering the development of better treatments [276]. All of these, combined with the efforts from international research groups and consortia, such as the TCGA or the ICGC, will provide new and better insights into the genetic characteristics of diverse types of cancer. Integrated data analysis is an important aspect of precision oncology research and has led to groundbreaking discoveries that would not have been possible without multi-omics analysis. Although various efforts have been made to develop machine learning-based methods to automate the integration levels of omics data [277], this is not a simple task. The approaches still need to solve problems such as batch effects and normalization within the integration analysis of the different types of data, and also be able to integrate different types of data, such as metabolomics data, which have shown to have a significant impact on cancer pathogenesis [278].

Optimal annotation and prioritization of variants is also a bottleneck in current precision oncology. This process requires databases containing curated variants as well as links with mechanistic effects and potential drug interaction data. However, simple and completely automated methods to carry out these processes are not yet available. In addition, large datasets of cancer patients, including their response to therapy, are needed so that effective machine-learning-based algorithms can be designed.

Long-read sequencing enables more comprehensive analysis of cancer genomes, solves complex genomic aberrations, improves the study of long transcript isoforms, and epigenomic modifications. The use of long reads in a multitude of cancer types is widespread, despite it not yet being leveraged in cutaneous melanoma. However, this technology still has several limitations to solve, including the higher error rates and the difficulty of obtaining high-molecular weight DNA material in sufficient quantities from the most common types of biobanked tumor samples [279].

## 8. Concluding Remarks

Because of the multiple advantages, the tremendous growth of applications, and the steep reductions in the cost per base, NGS has revolutionized research in melanoma and cancer genomics in the past decade. Despite the recent advances in the technology, including moving towards leveraging longer reads to optimally assess SVs, which are central in cancer, these improvements still lack a translation to the field. In order to continue pushing this field further, it will also be important to standardize the bioinformatics procedures and update the annotation databases with the most recent and complete references, as they will allow us to better identify somatic and germline sequence variation that is key in the pathogenesis of melanoma.

## Figures and Tables

**Table 1:** Overview of the most common commercial NGS platforms and instruments.

Brand	Instrument	Key Applications	Run Time (h)	Max. Output (Gb)	Max. Read Length (Bases)
Illumina, Inc.	NextSeq 550	Targeted Gene Sequencing Transcriptome Sequencing	12–30	120	PE150
	NextSeq 1000 and 2000	WGS (limited samples) WES Targeted Gene Sequencing Transcriptome Sequencing	11–48	360	PE150
	NovaSeq 6000	WGS WES Targeted Gene Sequencing Transcriptome Sequencing Methylation Sequencing	13–44 *	6000	PE250
	NovaSeq X Series	WGS (large sample number) WES Targeted Gene Sequencing Transcriptome Sequencing Methylation Sequencing	13–48 *	16,000	PE150
MGI Tech	DNBSEQ-G50	Targeted Gene Sequencing	9–40	150	PE150
	DNBSEQ-G400	WGS (limited samples) WES Transcriptome Sequencing	13–109 *	1440	PE300
	DNBSEQ-T7	WGS (large sample number) WES Targeted Gene Sequencing Transcriptome Sequencing	24–30	6000	PE150
Ion Torrent	Ion GeneStudio S5/Plus/Prime	WES (limited samples) Targeted Gene Sequencing	6–19	15/30/50	SE200/SE400/SE200
	Genexus System	WES (limited samples) Targeted Gene Sequencing	2–3	15	SE200

* Depends on the flow cell used. WES, whole-exome sequencing; WGS, whole-genome sequencing; Gb, gigabases; PE, paired-end; SE, single-end.

**Table 3:** Main long-read sequencing technologies or approaches and highlighted cancer genomics research.

Technology	Instruments	Read Characteristics	Related Somatic Studies
Oxford Nanopore Technologies	MinION GridION PromethION	Single molecule reads, average read length ~15–20 Kb (max ~2 Mb), with an error rate of 5–10%	Brain tumor [208], lung cancer [209]
Pacific Biosciences	Sequel Sequel II	HiFi reads, average read length ~15–20 Kb (max ~65 Kb), with error rate of 1%	Breast cancer [19]
Linked-reads (10x Genomics)	NextSeq HiSeq NovaSeq	Linked-reads obtained from short reads, average length ~100 Kb	Prostate cancer [210], gastric cancer [211]
Hi-C	NextSeq HiSeq NovaSeq	~1 kb–1 Mb resolution, without base pair resolution	Pancreatic cancer [212]
Optical maps (BioNano Genomics)	NextSeq HiSeq NovaSeq	Optical mapping of long fragments, average length 250 Kb, without base pair resolution	Leukemia [213]

Hi-C, chromosome conformation capture sequencing; Kb, kilobases; Mb, megabases.

**Table 4:** Common computational tools for long-read sequencing data analysis.

Bioinformatic Analysis	Tool	Sequencing Strategy	References
Base calling	Guppy, Bonito	ONT	https://github.com/nanoporetech/ (accessed on 2 August 2022)
	Generate CCS	PacBio	[242]
Quality control	pycoQC, NanoPack	ONT	[239,240]
	Isoseq3	PacBio	https://github.com/PacificBiosciences/IsoSeq (accessed on 2 August 2022)
Read-error correction	Canu	ONT	[243]
	LoRMA	PacBio	[244]
DNA methylation	pycoMeth, DeepSignal, Megalodon	ONT	[245,246]; https://github.com/nanoporetech/megalodon (accessed on 2 August 2022)
	pb-CpG-tools	PacBio	https://github.com/PacificBiosciences/pb-CpG-tools (accessed on 2 August 2022)
Alignment	minimap2, NGMLR	ONT	[117,247]
	pbmm2	PacBio	https://github.com/PacificBiosciences/pbmm2 (accessed on 2 August 2022)
SNV calling	Longshot, DeepVariant	ONT, PacBio	[248,249]
SV calling	Sniffles, SVIM, SVIM-asm, cuteSV	ONT	[247,250,251,252]
	pbsv	PacBio	https://github.com/PacificBiosciences/pbsv (accessed on 2 August 2022)
De novo assembly	Flye, Shasta	ONT	[253,254]
	Hifiasm, FALCON	PacBio	[255,256]
Hybrid assembly	MaSuRCA, WENGAN	ONT, PacBio	[257,258]
Polishing	Racon, Medaka, Pilon	ONT	[259,260]
	Pilon, Quiver, Arrow	PacBio	[260,261]

SNV, small nucleotide variant; SV, structural variant; ONT, Oxford Nanopore Technologies; PacBio, Pacific Biosciences.

## Data Availability

Not applicable.
